# Evaluation of a novel lower radiation computed tomography protocol for assessment of tunnel position post anterior cruciate ligament reconstruction

**DOI:** 10.1186/s12880-020-00480-5

**Published:** 2020-07-15

**Authors:** Varun Vasudeva, Stephen Key, Alfred Phillips, Steve Kahane, Joseph Stevens, Chris Wall, Price Gallie

**Affiliations:** 1 Gold Coast University Hospital, Gold Coast, Queensland Australia; 2 Toowoomba Hospital, Toowoomba, Australia

**Keywords:** ACL reconstruction, Tunnel position, CT scan

## Abstract

**Background:**

Anterior cruciate ligament (ACL) reconstruction is a common orthopaedic procedure. We developed a novel, low dose computed tomography (LDCT) protocol to assess tunnel position post-operatively. The effective radiation dose of this protocol is < 0.5millisieverts (mSv), which is significantly less than the 2 mSv dose for a conventional CT protocol. The aim of this study was to assess the accuracy of the LDCT protocol for determining tunnel position.

**Methods:**

Twenty-six patients who underwent primary ACL reconstruction were included in the study. A LDCT scan was performed 6 weeks post-operatively. Femoral and tibial tunnel positions were measured on three dimensional (3D) reconstructions using previously validated techniques. Measurements were performed independently by three observers at two time points, 4 weeks apart.

**Results:**

There was excellent intra- and inter-rater reliability for all measurements using the images obtained from the LDCT protocol. Intra-class correlation coefficient (ICC) values were > 0.9 for all measurements.

**Conclusions:**

The LDCT protocol described in this study accurately demonstrates femoral and tibial tunnels post ACL reconstruction, while exposing the patient to a quarter of the radiation dose of a conventional CT. This protocol could be used by orthopaedic surgeons for routine post-operative imaging, in place of plain film radiographs.

## Background

Rupture of the ACL is a common sporting injury [1]. ACL reconstruction is an effective orthopaedic procedure, allowing return to sporting activity after appropriate rehabilitation [2]. However, graft rupture is a common complication, occurring in up to 20% of cases, with younger patients being at higher risk [3]. A significant portion of graft ruptures may be attributed to technical error, specifically with improper bone tunnel placement [4, 5]. Suboptimal tunnel position results in increased risk of symptomatic knee instability and graft rupture, independent of graft type or tunnel preparation technique [6].

Tunnel position may be assessed post operatively using a number of imaging modalities. Plain film radiography is commonly used, with anteroposterior (AP) and lateral x-rays of the knee demonstrating the bony tunnels. A number of techniques have been proposed to determine correct tunnel position [7]. However, x-rays only provide a two-dimensional (2D) image and hence may be difficult to interpret [7]. Computed tomography (CT) with 3D image reconstruction allows detailed assessment of osseous anatomy and has been shown to accurately demonstrate tunnel position [8, 9]. However, CT has the disadvantage of a higher radiation dose to the patient. We developed a novel LDCT protocol, with the goal of accurately assessing tunnel position post ACL reconstruction, while minimising radiation exposure to the patient. The purpose of this study was to evaluation the accuracy of this protocol in determining the femoral and tibial tunnel positions.

## Methods

Patients undergoing isolated primary ACL reconstruction by the senior author (PG) over a 12-month period were prospectively recruited to the study. Paediatric patients (aged less than 18 years) and patients undergoing revision surgery were excluded. Ethics approval was obtained from our local research ethics committee, and all patients provided informed consent prior to inclusion in the study.

A single bundle ACL reconstruction was performed with a four-strand short graft, using the Tape Lock Screw technique (TLS®, FH Orthopaedics, Heimsbrunn, France) [10]. This technique utilises the ipsilateral semitendinosus tendon, which is fashioned into a four-strand closed loop (50-60 mm in length, depending on height and gender) and pre-tensioned to 500 N for 1 minute. Femoral and tibial tunnels are created independently in a retrograde manner to match the diameter of each end of the graft. The tunnels are centred over the midpoint of the anatomic footprints of the native ACL. The graft is introduced using an all-inside technique and secured with polyethylene terephthalate tapes passed through each end of the closed tendon loop and suspended in the bone tunnels with interference screws.

**Fig. 1:**
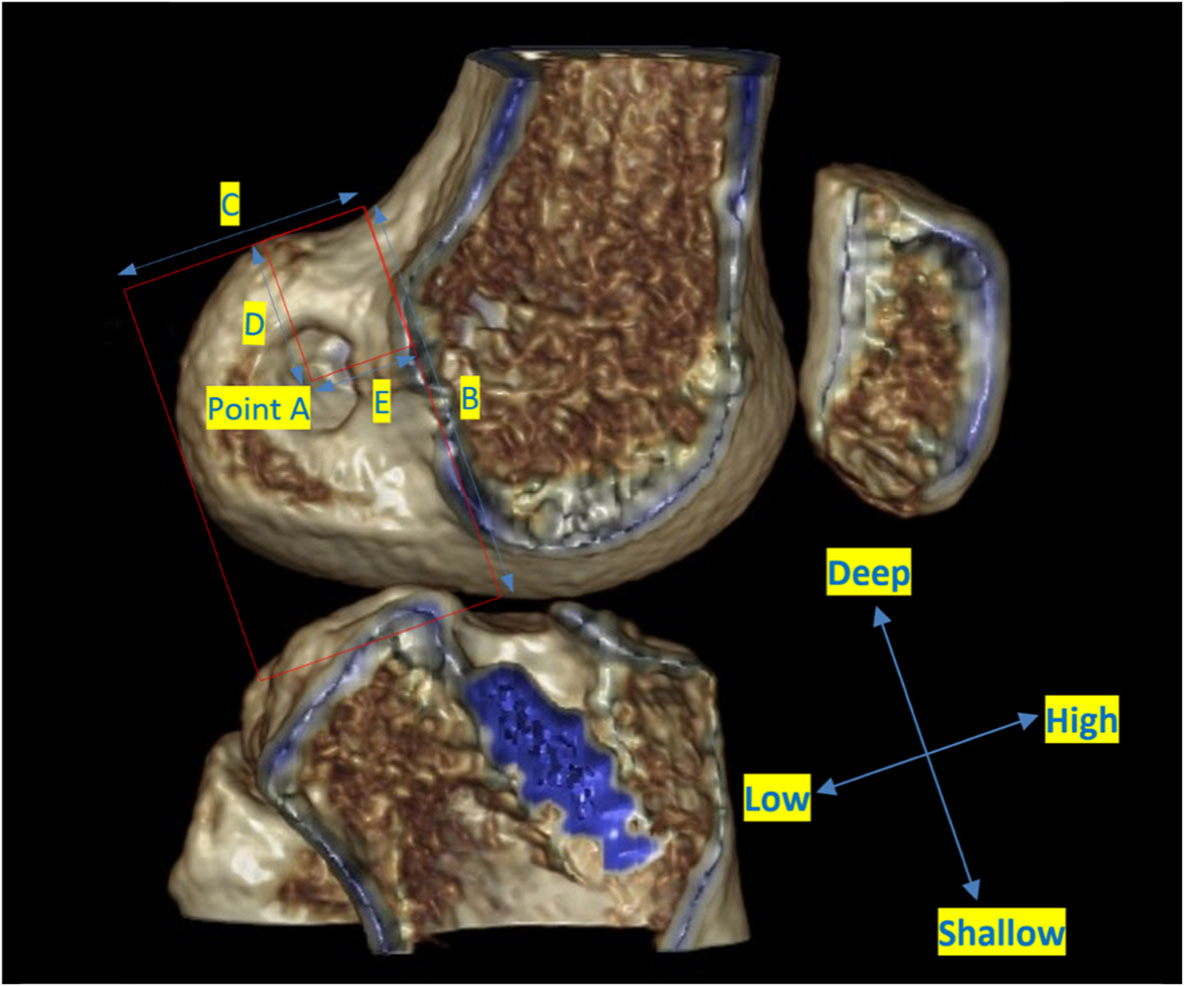
3D femoral reconstruction showing quadrant method to assess tunnel position

**Fig. 2:**
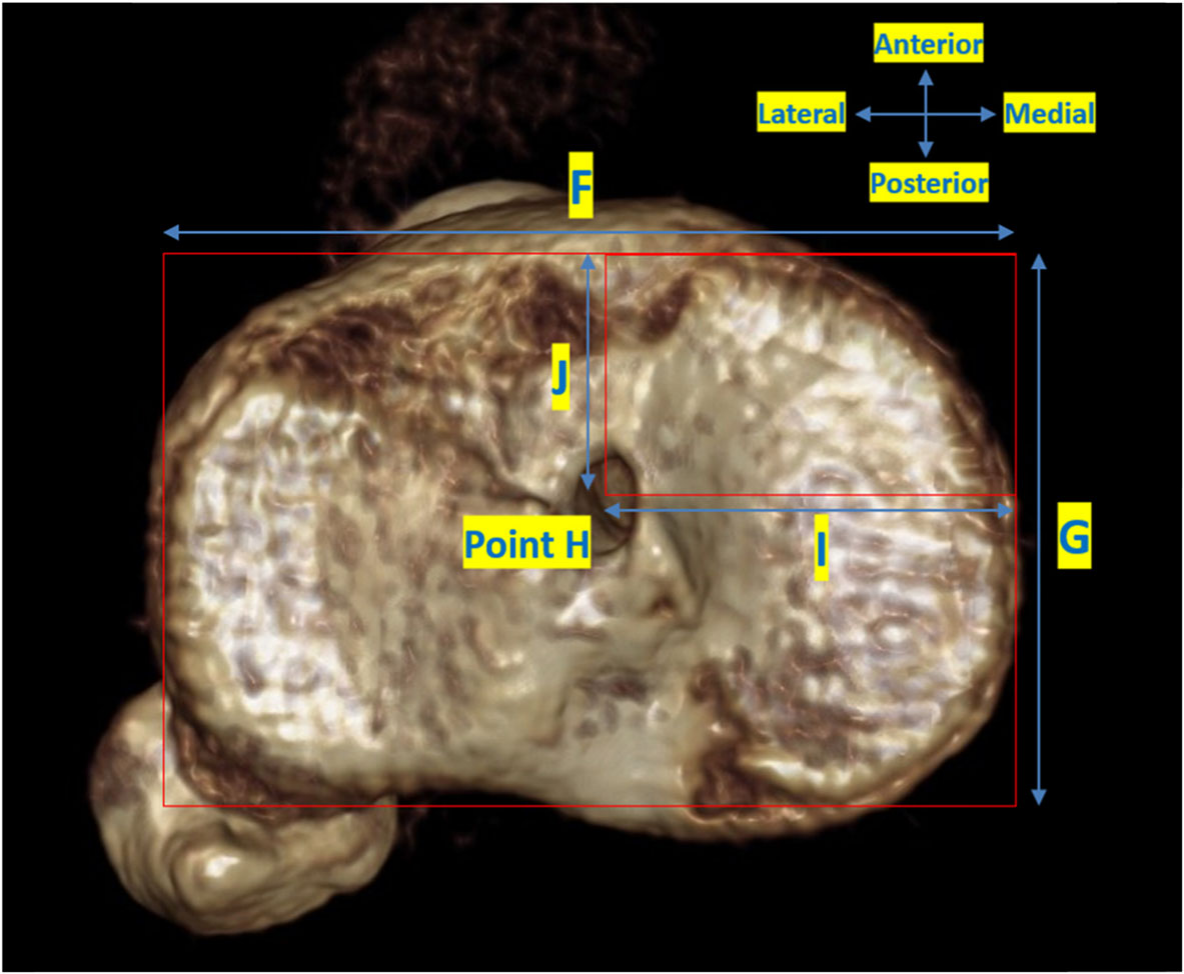
3D tibial reconstruction showing rectangular reference frame to assess tunnel position

All patients underwent a standardised LDCT 6 weeks post operatively. A Siemens Definition AS+ CT scanner (Siemens Somatom Definition AS+; Siemens Healthcare, Forchheim, Germany) was used. The data was then processed through the clinical software application iterative Metal Artifact Reduction (iMAR) algorithm for metal artifact reduction. Data was imported into Philips 2nd edition software (Philips Medical Systems North America, Shelton, Conn) to create a complete 3D volume reconstruction of each femur (to 10 cm above the femoral condyle) and tibia (to 1 cm below the tibial tubercle). The femoral 3D reconstruction had at least 90% overlap of femoral condyles and then virtual subtraction of the medial femoral condyle at the highest point of the intercondylar notch, leaving the most medial sagittal aspect of lateral femoral condyle with tunnel position on view (Fig. 1). The tibial 3D reconstruction was an axial view, adjusted to view the most superior aspect of the proximal tibia with the femur and patella removed (Fig. 2). The 3D reconstructions were imported into IntelliSpace Patient Archiving and Communication System (IntelliSpace PACS Enterprise; Philips) for measurement.

### Radiation dose and associated risk

The LDCT protocol used the following technique factors; 80kVp, 80mAs (effective), 128 × 0.6 mm collimation, and helical scan with 1 mm slice reconstructions. The scan coverage was approximately 10 cm from above the femoral condyles to 1 cm below the tibial plateau. The organ and effective dose applicable to research participants was estimated using three methods.

The first method was estimated using the International Commission on Radiological Protection 103 methodology (Table 1) [11]. This method used the assumption that the radiosensitive organs within the irradiation area (red bone marrow, bone surface and skin mass) constitutes less than 10% of the total mass of each of these organs in the body. This assumption was based on a conservatively estimated irradiation bone mass of less than 500 g (i.e. 300cm^3^ × 1.65 g/cm^3^) and a total body bone mass greater than 5 kg [12]. The percentage of irradiated skin compared to total skin would be significantly less than 10%. The organ dose for radiosensitive organs was estimated using the central axis CT Dose Index (CTDI), where CTDI free-in-air would offer a maximum delivered dose to any point within the irradiated region. It was assumed that, given the relatively small cross-sectional area of the irradiated anatomy, organ doses would be approximately equal to, or slightly less than this value. The CTDI free-in-air value was taken from measurements of the Siemens Definition AS model outputs for technique factors matched as closely as possible to those proposed for this study [13]. This was calculated to be 7.8 mGy/100mAs (Table 2).

**Table 1:** Method 1 - Organ and effective dose calculation

Organ	Total Weighting Factor	Dose Equivalent (μSv)	w.f. x dose equivalent (μSv)
Colon, lung, stomach, breast	*0.48*	NA	NA
Bladder, liver, thyroid	*0.16*	NA	NA
Bone-marrow	*0.24*	7800 × 0.1	188
Gonads	*0.08*	NA	NA
Bone surface, skin	*0.02*	7800	16
Brain	*0.02*	NA	NA
Total (effective dose)	*1.0*	NA	< 500

**Table 2:** Method 2 - Organ and effective dose summary

Organ	Dose
Red bone marrow, bone surface, skin	*0.78 mGy*
Effective dose (whole body)	*< 0.5 mSv*

**Fig. 3:**
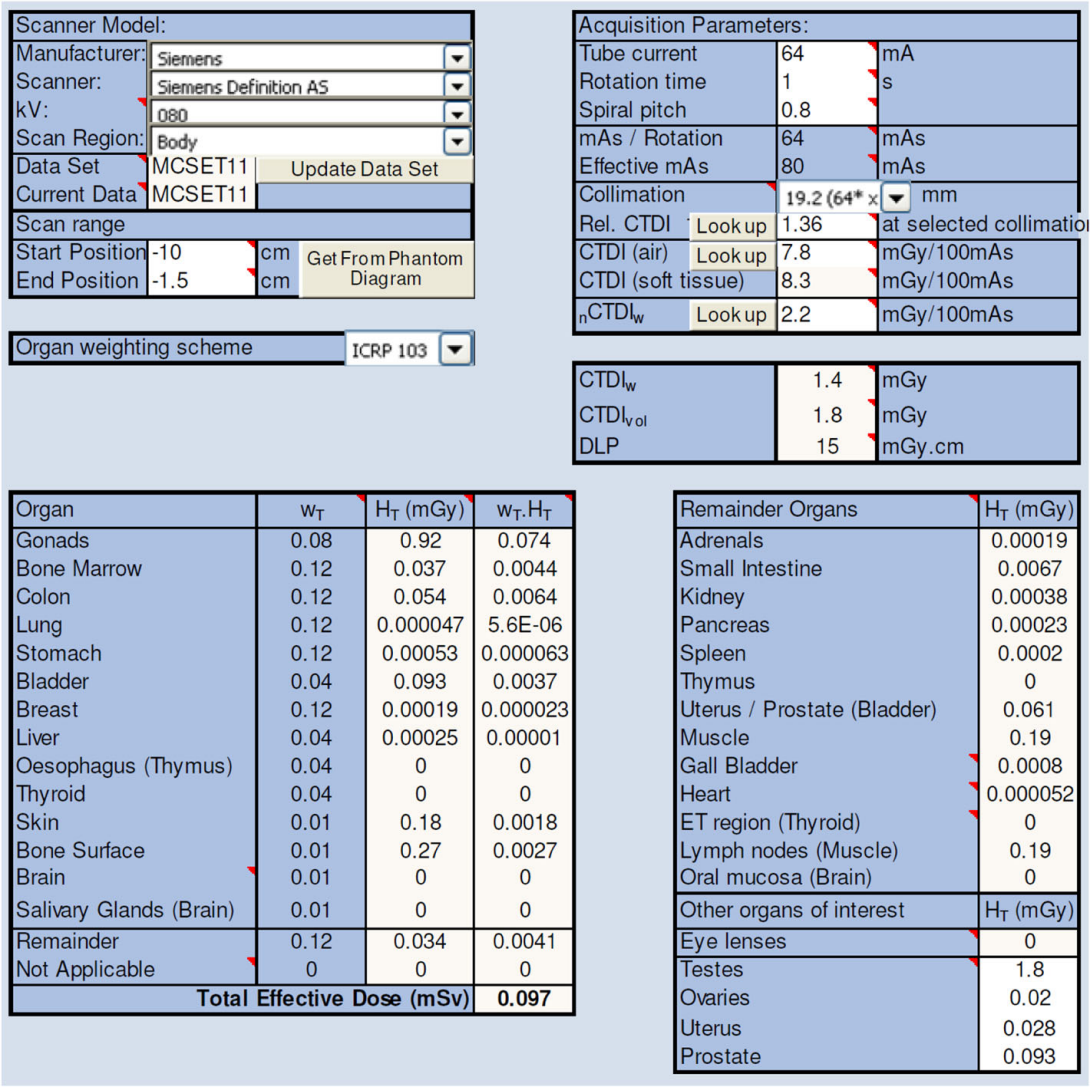
Patient Dosimetry Calculator

The second method was estimated using the Impact CT dose program which bases its data on the Monte Carlo modelling of the National Radiological Protection Board (NRPB) [14]. This calculation used the deposition of energy at the proximal femur to estimate organ and effective doses for a scan distal to the knee. Based on this model and taking into account the estimates used in method one, the organ and effective doses (with the assumption an average sized patient is 70 kg) for the LDCT scan, calculations can be seen in Fig. 3 and are summarised in Table 3.

**Table 3:** Method 3 - Organ and effective dose summary

Organ	Dose
Red bone marrow, bone surface, skin	*< 0.5 mGy*
Effective dose (whole body)	*< 0.1 mSv*

The third method used recently published data on Monte Carlo modelling of radiation transport from current generation CT scanners through voxel-based digital phantoms has provided a conversion factor (k-factor) to convert the Dose-Length Production (DLP) obtained from a given CT scanner to an effective dose value [15]. The k-factor derived for an 80kVp CT scan of an adult male or female was reported to be 0.0004. With a conservatively estimated DLP for this scan of 50 mGy, the effective dose estimated using method would be < 0.1 mSv. Based on the above estimations and methods, the total effective dose for a single procedure is < 0.5 mSv.

### Femoral tunnel measurement

The quadrant method described by Bernard was used to assess femoral tunnel position (Fig. 1) [11, 16]. Point A is the centre of the femoral tunnel on the medial aspect of the lateral femoral condyle. A rectangular reference frame is superimposed. Distance B is the total sagittal diameter of the condyle, measured along the intercondylar notch roof, limited by the shallowest and deepest contours of the condyle. Distance C is the height of the intercondylar space, measured as the perpendicular distance between the notch roof and a parallel line tangential to the lowest point on the femoral condyle. Distance B and Distance C define the intercondylar space and create the axes for the quadrant system. Distance D is measured between Point A and the deep contour of the condyle, parallel to the notch roof. Distance E is the perpendicular distance between Point A and the notch roof. Femoral tunnel position in the sagittal plane is the defined by calculating ratios D/B and E/C.

### Tibial tunnel measurement

The tibial tunnel position was assessed using a rectangular reference frame, as has been previously described (Fig. 2) [11, 17]. The posterior border is drawn tangential to the posterior margins of the medial and lateral articular surfaces. The anterior border is drawn parallel to the posterior border, tangential to the anterior margin of the medial articular surface. The medial and lateral borders are drawn tangential to the most medial and lateral articular margins, respectively, perpendicular to the posterior border. Distance F is the mediolateral (ML) dimension of the tibial plateau. Distance G is the AP dimension of the tibial plateau. Point H is the centre of the tibial tunnel. Distance I and Distance J are measured perpendicularly from the medial and anterior borders of the reference frame, respectively. Tibial tunnel position is then defined in the axial plane by calculating ratios I/F and J/G.

### Statistics

Femoral and tibial tunnel position was measured for each case independently by three observers (VV, SKe, SKa) at two time intervals, 4 weeks apart, in a blinded fashion. Images were de-identified and presented in random order, and the sequence was changed for the second measurement. Statistical analysis was performed using SPSS version 23.0 software (IBM Corp. IBM SPSS Statistics for Windows, Armonk, New York). Intra- and inter-rater reliability was calculated using a 2-way random absolute agreement model ICC and standard error of measurement (SEM). Single measures ICC was used to determine intra-rater reliability and average measures ICC was used to determine inter-rater reliability by comparing the means of 2 measurements of each variable.

## Results

A total of 26 patients were included in the study. There were 15 females and 11 males with a mean age of 31 years (range 20 to 47 years). Femoral and tibial tunnel measurements and intra- and inter-rater ICC values are presented in Table 4. There was excellent intra- and inter-rater agreement for all measurements (ICC > 0.9) using the LDCT protocol. Active scan lengths on average were 7 seconds. The effective radiation dose of the LDCT protocol was measured as < 0.5 mSV and the expected uterine equivalent dose was < 0.1 mSv. This compares to an average 1.99 mSv dose for a standard knee CT using the scanner at our institution.

**Table 4:** Measurements of femoral and tibial tunnel position and ICC values

	Femoral		Tibia	
	*D/B*	E/C	J/G	I/F
Mean	0.337	0.270	0.437	0.468
SD	0.077	0.083	0.063	0.026
Min	0.154	0.086	0.218	0.389
Max	0.500	0.506	0.590	0.534
Inter-rater				
ICC	0.995	0.99	0.964	0.961
Lower bound (95% CI)	0.980	0.974	0.932	0.923
Upper bound (95% CI)	0.998	0.996	0.983	0.982
SEM	0.005	0.008	0.012	0.005
Intra-rater 1				
ICC	0.965	0.922	0.931	0.936
Lower bound (95% CI)	0.925	0.823	0.852	0.863
Upper bound (95% CI)	0.984	0.965	0.968	0.971
SEM	0.014	0.023	0.016	0.007
Intra-rater 2				
ICC	0.972	0.921	0.949	0.953
Lower bound (95% CI)	0.939	0.821	0.89	0.899
Upper bound (95% CI)	0.987	0.965	0.977	0.979
SEM	0.013	0.023	0.014	0.006
Intra-rater 3				
ICC	0.969	0.96	0.968	0.923
Lower bound (95% CI)	0.929	0.915	0.931	0.837
Upper bound (95% CI)	0.986	0.982	0.986	0.964
SEM	0.013	0.017	0.011	0.007

## Discussion

ACL reconstruction is a well-established treatment for ACL deficient patients, and the incidence of the procedure is increasing [1]. Surgeons often obtain post-operative imaging to assess the femoral and tibial tunnel positions. Plain film radiography and CT are the two commonly used modalities, and each has advantages and disadvantages. Plain film radiography is used by many surgeons to assess tunnel position, given its ease of access, low cost, and low radiation exposure to the patient [14]. However, several studies have shown that CT scans, especially with 3D reconstruction, more accurately demonstrate the bony tunnels than standard orthogonal knee x-rays following primary ACL reconstruction [12, 18]. Similarly, when considering revision ACL reconstruction, CT scans have also demonstrated superiority to plain radiographs in accurately assessing tunnel position [13]. CT imaging is becoming more readily available, however does come at the cost of a higher radiation dose to the patient [19].

Reported effective radiation doses for knee CT vary widely in the literature [20–22]. This likely reflects variations in equipment, protocols and intended applications. At our institution, a standard knee CT protocol exposes the patient to an average effective radiation dose of 1.99 mSv. Similarly, the effective radiation dose for orthogonal knee x-rays will vary from hospital to hospital [23]. At our institution, standard orthogonal knee x-rays expose the patient to an effective radiation dose of approximately 0.6 μSv.The LDCT protocol presented in this study was developed to allow 3D measurement of bony tunnel position while minimizing radiation exposure to the patient. The effective radiation dose was measured at < 0.5 mSv, which is a quarter of the radiation dose of a conventional CT. Despite the low radiation dose, this protocol allows accurate and reproducible 3D reconstructions with reliable visualisation of the femoral and tibial tunnels. This was demonstrated by the high intra- and inter-rater reliability of tunnel position measurements (ICC > 0.9 for all measurements). No harmful effects of radiation exposure of < 0.5 mSv have been demonstrated [19]. The Australian Radiation Protection and Nuclear Safety Agency (ARPANSA) recommend no more than 5 mSv exposure per year [24]. The theoretical risk of cancer incidence for an exposure of 0.5mSV using age and sex specific factors in a 20-year-old male and female is 1:20,000 and 1:12,000 respectively [19]. The theoretical cancer risk decreases with increasing patient age.

The major strength of this study is its applicability to clinical practice. All measurements were performed in CT scans of real patients performed post-operatively, with hamstring grafts and fixation devices in situ. This is in contrast to previously described cadaveric models [11]. We have demonstrated that a high degree of accuracy can be achieved using this protocol in a clinical setting. Furthermore, this study demonstrates that a high degree of accuracy can be achieved with this LDCT protocol by observers at different levels of surgical training. Two of the observers in this study were orthopaedic surgeons and one was a junior orthopaedic registrar. A high degree of inter-rater reliability was demonstrated for all measurements, despite the observers’ different levels of training. This LDCT protocol may be instituted as standard post-operative imaging as detection of sub-optimal tunnel position may be recognised earlier and more accurately, thereby directly affecting clinical management.

A single validated method was chosen to describe each bony tunnel, as the goal of this study was to determine the reliability of the LDCT protocol, rather than to evaluate the measurement methods themselves. Despite the quadrant method for femoral tunnel position originally being described for plain film radiographs, multiple studies have demonstrated high levels of intra- and inter-rater reliability when applied to 3D CT reconstructions [11, 25]. The current study supports these findings. Similarly, the described method for defining tibial tunnel placement has also been shown to be highly reliable in previous studies, as well as the current study [25]. Our results are comparable with those using standard CT protocols, indicating that the LDCT protocol is reliable for determining bony tunnel position [11, 17].

We acknowledge that this study has limitations. The sample size of 26 patients is small, however this is comparable with previously published studies validating tunnel position measurement protocols [8, 11, 26]. One of the limitations of this study is the lack of a control group. A retrospective control group was not available, as our previous practice had been the use of post-operative plain film radiography to assess tunnel position. The standard CT radiation dose was calculated based on studies performed on patients with failed ACL reconstructions. We considered including a prospective control group, however felt we could evaluate the LDCT protocol without subjecting patients to standard dose CT scans. Previous studies have validated the measurement methods we used to determine femoral and tibial tunnel positions using standard CT protocols. Our outcome of interest was the accuracy of tunnel position measurement using the LDCT protocol, which we have clearly demonstrated. We felt that subjecting control patients to a higher radiation exposure was unjustified [8, 17]. The surgical technique used in this study attempts to place the ACL graft in an anatomic position, using the native femoral and tibial footprints as landmarks. It is possible that reconstruction techniques using alternative tunnel positions may not be reliably assessed using this LDCT protocol. Alternative 3D reconstructions may be required in such cases, and we cannot comment on the reliability of this protocol to demonstrate the tunnel positions.

## Conclusion

The novel LDCT protocol described in this study provides 3D images which accurately demonstrate the bony anatomy of femoral and tibial tunnels following ACL reconstruction, while minimizing radiation exposure to the patient. We demonstrated excellent intra- and inter-rater reliability using previously validated measurement techniques. The radiation exposure to the patient using this LDCT protocol is a quarter of that experienced with a conventional CT. We believe this LDCT protocol could be used by orthopaedic surgeons for routine post-operative imaging of ACL tunnel position, in place of plain film radiographs.

## Data Availability

Data images and materials are available for further viewing and review if required by request of the corresponding author on reasonable request.

## Abbreviations

ACL: Anterior cruciate ligament
mSv: Millisieverts
LDCT: Low dose computed tomography
3D: Three dimensional
ICC: Intra-class correlation coefficient
AP: Anteroposterior
CT: Computed tomography
iMAR: Iterative Metal Artifact Reduction
CTDI: CT Dose Index
NRPB: National Radiological Protection Board
DLP: Dose-Length Production
ML: Mediolateral
SEM: Standard error of measurement
ARPANSA: Australian Radiation Protection and Nuclear Safety Agency
